# The Pathology of Severe Dengue in Multiple Organs of Human Fatal Cases: Histopathology, Ultrastructure and Virus Replication

**DOI:** 10.1371/journal.pone.0083386

**Published:** 2014-04-15

**Authors:** Tiago F. Póvoa, Ada M. B. Alves, Carlos A. B. Oliveira, Gerard J. Nuovo, Vera L. A. Chagas, Marciano V. Paes

**Affiliations:** 1 Laboratório de Biotecnologia e Fisiologia de Infecções Virais, Instituto Oswaldo Cruz, Fundação Oswaldo Cruz, Rio de Janeiro, Brazil; 2 Hospital Universitário Gaffrée Guinle, Departamento de Anatomia Patológica, Universidade Federal do Estado do Rio de Janeiro, Rio de Janeiro, Brazil; 3 University Comprehensive Cancer Center, Columbus, Ohio, United States of America; 4 Hospital Universitário Clementino Fraga Filho, Departamento de Anatomia Patológica, Universidade Federal do Rio de Janeiro, Rio de Janeiro, Brazil; University of Rochester, United States of America

## Abstract

Dengue is a public health problem, with several gaps in understanding its pathogenesis. Studies based on human fatal cases are extremely important and may clarify some of these gaps. In this work, we analyzed lesions in different organs of four dengue fatal cases, occurred in Brazil. Tissues were prepared for visualization in optical and electron microscopy, with damages quantification. As expected, we observed in all studied organ lesions characteristic of severe dengue, such as hemorrhage and edema, although other injuries were also detected. Cases presented necrotic areas in the liver and diffuse macro and microsteatosis, which were more accentuated in case 1, who also had obesity. The lung was the most affected organ, with hyaline membrane formation associated with mononuclear infiltrates in patients with pre-existing diseases such as diabetes and obesity (cases 1 and 2, respectively). These cases had also extensive acute tubular necrosis in the kidney. Infection induced destruction of cardiac fibers in most cases, with absence of nucleus and loss of striations, suggesting myocarditis. Spleens revealed significant destruction of the germinal centers and atrophy of lymphoid follicles, which may be associated to decrease of T cell number. Circulatory disturbs were reinforced by the presence of megakaryocytes in alveolar spaces, thrombus formation in glomerular capillaries and loss of endothelium in several tissues. Besides histopathological and ultrastructural observations, virus replication were investigated by detection of dengue antigens, especially the non-structural 3 protein (NS3), and confirmed by the presence of virus RNA negative strand (*in situ* hybridization), with second staining for identification of some cells. Results showed that dengue had broader tropism comparing to what was described before in literature, replicating in hepatocytes, type II pneumocytes and cardiac fibers, as well as in resident and circulating monocytes/macrophages and endothelial cells.

## Introduction

Dengue infection is the most prevalent arthropod-borne viral disease in subtropical and tropical regions of the world. The dengue virus (DENV) belongs to the *Flaviviridae* family and consists of four antigenically distinct serotypes (DENV1-4). The infection can result in a broad spectrum of effects, including acute febrile illness, the dengue fever (DF), which may progress to severe forms such as dengue hemorrhagic fever (DHF) and dengue shock syndrome (DSS), with changes in hemostasis and vascular permeability [1], [2]. Several studies indicate that the occurrence of secondary infection with a heterologous serotype increase the risk of developing DHF [3], [4]. Therefore, in areas where multiples DENV serotypes circulate, such as in Brazil, sequential infections may occur, which lead to the increase in the number of severe dengue cases [5]–[7]. Moreover, other risk factors, such as ethnicity, age, co-morbidities, genetic predisposition and immune conditions of the patient, as well as genetic variations of viral strains, may also contribute for the occurrence of DHF [8]–[10]. Severe dengue disease is characterized by circulatory damages, associated in most cases with hepatic dysfunctions [11]–[13]. These injuries may be a direct consequence of the virus presence and/or resulted by an exacerbation of the immune response after infection [14], [15].

Overall, *in vivo* studies regarding DENV infection and its pathogenesis are limited by the lack of an experimental animal model able to mimic the full spectrum of the disease as observed in humans [16]. Therefore, there are still several gaps in understanding the pathogenesis of dengue. On the other hand, autopsy studies based on human dengue cases are extremely important and may clarified some of these gaps, pointing out for example how and which tissues are affected during the disease. Most of histopathological reports with dengue human fatal cases indicate that the liver, spleen and lymph nodes are target organs of infection [17]–[19]. Besides the occurrence of hemorrhage and edema in the liver of dengue fatal cases, histopathological analysis also reported damages caused by metabolic alterations and/or inflammatory reactions, such as the presence of steatosis, areas with infiltrated cells and necrosis and hyperplasia and destruction of Kupffer cells [17], [18], [20]–[22]. Additionally, other studies showed several lesions in spleen tissues, such as interstitial edema, vascular congestion, splenic rupture and bleeding [17], [18], [23]. However, recently, atypical clinical manifestations of dengue have been reported, involving the kidney, lung, heart and central nervous system, which were also corroborated by histopathological findings revealing several areas with hemorrhage, edema and inflammatory infiltrates in these organs [24]–[28].

In addition to histopathological analysis, very little is known about the ultrastructural aspects of affected organs in dengue human cases. In fact, as far as we know, the only study described in the literature with electron microscopy evaluation is from Limonta et al. [29], showing the presence of virus-like particles in neuroglia, hepatocytes and alveolar and splenic macrophages in one human fatal case.

Therefore, in the present work we characterized histopathological and ultrastructural aspects of the liver, lung, heart, kidney and spleen of four DENV-3 fatal cases occurred in Rio de Janeiro, Brazil, in the dengue outbreak from 2002. These patients developed DHF with several dysfunctions in all the studied organs. Virus tropism and replication was also evaluated, by immunohistochemical and *in situ* hybridization analysis, revealing the presence of the virus in different cells, such as resident and circulating monocytes/macrophages, endothelial cells, hepatocytes, type II pneumocytes and cardiac fibers. Results showed that this virus had a broader tropism comparing to what was described before in literature, leading to drastic lesions in several organs.

## Materials and Methods

### Ethical Procedures

All procedures performed during this work were approved by the Ethics Committee of the Oswaldo Cruz Foundation/FIOCRUZ, with the number 434/07 for studies with fatal dengue cases and controls. The institutional review board or ethics committee waived the need for consent.

### Human Fatal Cases

The human tissues analyzed in this study (liver, lung, heart, spleen and kidney) were obtained from four dengue fatal cases in 2002 in Rio de Janeiro. During the summer of 2002, Rio de Janeiro had a large epidemic of DF and DHF and 99% of cases were caused by DENV-3 [30]. Our cases were patients admitted in the Hospital São Vicente de Paulo (case 1), Hospital Universitário Clementino Fraga Filho (case 2) and the Hospital Miguel-Couto (cases 3 and 4). The available information of clinical and necropsy data concerning the four fatal cases is listed below and described in Table 1. All patients died with a clinical diagnosis of dengue hemorrhagic fever, with classical symptoms (fever, myalgia and hemorrhagic manifestations). Necropsy revealed that cases presented extensive areas of hemorrhage and edema in all analyzed organs. The dengue diagnosis was confirmed by positive serum IgM antibodies. The four negative controls, from both sexes and ranging from 40 to 60 year old, were non-dengue or any other infectious disease case.

**Table 1:** Hospital and necropsy records from the four dengue fatal cases.

Dengue cases:	1	2	3	4
**Age:**	63 years	21 years	41 years	61 years
**Sex:**	male	female	female	female
**Symptoms:**	fever	fever	fever	fever
	myalgia	myalgia	weakness	myalgia
	headache	diarrhea	fainting	vomiting
	anorexia	nausea	sweating	diarrhea
	Rash	vomiting	abdominal pain	hypohydrated
	Pain	metrorrhagia	discharge yellow	
	diarrhea		epigastric pain	
	petechiae			
	bledding			
	hemoptysis			
**Laboratory workup:**	leukopenia	leukopenia	leukocytosis	–
	thrombocytopenia	thrombocytopenia		
	hemoconcentration			
**Necropsy data:**	ischemic myocarditis	alveolar edema	pulmonary edema	edema pulmonary
		hepatic steatosis	cardiomyopathy	cardiomyopathy
		periportal hepatitis	ischemia	hepatic congestion
		splenic congestion	luteal cysts	pyelonephritis
				renal detention
**Co-morbities:**	diabetes	obesity	–	–
**Days of hospitalization:**	3 days	1 day	3 hours	5 days
**Confirmed diagnosis:**	IgM positive	IgM positive	IgM positive	IgM positive
	IgG positive	ND	ND	ND
	PCR positive	ND	ND	ND

ND: not determined.

### Case Presentation

#### Case 1

**Clinical data:** A 63-year-old male patient with diabetes mellitus, taking acetylsalicylic acid (100 mg) and Daonil, developed a sudden onset of headache, myalgia, anorexia, abdominal pain. Four days later he presented diarrhea, hemoptysis, leukopenia, thrombocytopenia (platelet 79.000/mm3) and hemoconcentration (hematocrit 59%). Biochemical parameters evaluated in the serum: aspartate aminotransferase 75 IU/L; alanine aminotransferase 21 IU/L; creatine phosphokinase 126 IU/L; creatinine 5.0 mg/dL; urea 57 mg/dL; lipase 41 IU/L. Ultrasonography revealed peri-hepatic and peri-pancreatic collections confirmed by computed tomography of the abdomen that also revealed: increase of the heart, discrete opacities in the left lung with marginal pleural reaction at the base of this hemithorax, distended gall bladder. Furthermore, he was submitted to routine of acute abdomen that revealed no gaseous distention. Physical examination on admission revealed blood pressure of 140/80 mmg and dehydration with cutaneous rash and petechiae. The patient presented a progressive worsening of the clinical, evolving to shock with severe pulmonary congestion, orotracheal intubation and respiratory orthesis, with the use of dopamine and dobutamine. In the next day, it was observed a hemodynamic instability and oliguria, with consequently administration of norepinephrine in increasing doses, culminating in refractory shock and death. The patient died with a clinical diagnosis of dengue hemorrhagic fever, severe ischemic cardiomyopathy and pancreatitis.

**Necropsy data:** Multiple purpuric and petechial hemorrhagic lesions were evident, especially around needle puncture sites. Hemorrhages were also present in serous cavities, including hemorrhagic pleuritis (sulphation of the visceral pleura) and fibrin pericarditis. The liver and spleen were grossly congested with multiple hemorrhagic foci, and a perforated duodenal ulcer was described.

#### Case 2

**Clinical data:** A 21-year-old female patient who experienced fever, myalgia and headache for 8 days and symptoms progressed to metrorrhagia, nausea, vomiting and diarrhea. Before hospitalization, she was examined in another health service with a hypothesized dengue diagnosis due to severe leukopenia and thrombocytopenia (platelet 10.000/mm3). The patient was admitted in the intensive care unit (ICU) at the Hospital Universitário Clementino Fraga Filho with respiratory failure, followed by the evolution of multiple organ failure and refractory shock. Biochemical parameters evaluated in the serum: aspartate aminotransferase 149 IU/L; alanine aminotransferase 66 IU/L; glucose 158 mg/dL; creatinine 1.10 mg/dL; urea 9.0 mg/dL.

**Necropsy data:** Ecchymosis, conjunctival hemorrhage, bilateral pleural effusion, pulmonary hemorrhage, renal congestion and splenomegaly.

#### Case 3

**Clinical data:** A 41-year-old female patient reporting fever for 2 days, weakness, fainting, sweating, yellow discharge, epigastric and abdominal pain. Laboratory workup demonstrated hematocrit of 48% and leukocytosis. Ultrasound showed fluid in the abdominal cavity. Patient died with a clinical diagnosis of dengue hemorrhagic fever causing an acute pulmonary edema (*causa mortis*).

**Necropsy data:** The autopsy revealed a presence of tracheal hyperemia, externally reddish wall of the duodenum, esophageal mucosa with irregular dark wall, pulmonary edema, bilateral pleural effusion, hypertrophic cardiomyopathy and yellowish brown myocardium, mild retroperitoneal hemorrhage, visceral polycongestion, ascites, yellowish hepatic parenchyma and spleen with diffluent parenchyma.

#### Case 4

**Clinical data:** A 61-year-old female hospitalized with suspected dengue symptoms (fever, myalgia, vomiting and diarrhea). Biochemical parameters evaluated in the serum: creatinine 1.07 mg/dL; urea 22.9 mg/dL; glucose 104 mg/dL. The patient died by acute pulmonary edema with sudden cardiac arrest.

**Necropsy data:** Cyanosis of the extremities, bilateral pleural effusion, bilateral pulmonary edema, pericardial effusion, hyperemia of the tracheal mucosa, yellowish streaks in thoracic aorta, hypertrophic cardiomyopathy, diffuse hyperemia in gastrointestinal tract, chronic cholecystitis, passive hepatic congestion, polyvisceral congestion, chronic pyelonephritis, renal retention cyst and mild cerebral edema.

### Histopathological Analyzes

Tissues samples from the human necropsies were fixed in formalin (10%), blocked in paraffin resin, cut in 4 µm, deparaffinized in xylene and rehydrated with alcohol, as described elsewhere [31]. Sections were stained with hematoxylin and eosin for histological examination and visualized in a Nikon ECLIPSE E600 microscope. Hemorrhage and edema, diffuse in the entire organs (liver, lung, heart, kidney and spleen) in all dengue cases, were analyzed quantitatively, using a millimetric ocular lens which allows us to calculate the percentage of injury tissues (areas of damage divided by the total area of the tissue in each slide). In the liver, steatosis, present in all hepatic lobules, was evaluated and quantified using a scale ranging from 0 to 4, according to the extensive of affected areas. Grade 0 was attributed to less than 1% of total affected hepatocytes, grade 1 between 1% and 25%, grade 2 between 25% and 50%, grade 3 between 50% and 75% and grade 4 more than 75% of affected hepatocytes, as described elsewhere [32]. A total of 40 fields of the dengue cases and controls (10 images for each case representing different lobules) were photographed at magnification of 400x and counted. Damages were characterized in the three areas of the hepatic acini (periportal, midzonal and central vein area) similar as performed by Quaresma et al. [33]. All analyzes were accomplished in a blind test without prior knowledge of the group.

### Immunohistochemistry Procedure

For immunohistochemical studies the paraffin-embedded tissues were cut (4 µm), deparaffinized in xylene and rehydrated with alcohol. Antigen retrieval was performed by heating the tissue in presence of citrate buffer [34]. Such tissues were then blocked for endogenous peroxidase with 3% hydrogen peroxidase in methanol and rinsed in Tris-HCl (pH 7.4). To reduce non-specific binding, sections were incubated Protein Blocker solution (Spring Biosciense) for 5 min at room temperature. Samples were then incubated over-night at 4°C with anti-DENV-3 polyclonal antibodies (raised in Swiss mouse inoculated with DENV-3), diluted 1∶300 in Tris-HCl, or with antibodies specific to recombinant dengue NS3 protein (expressed in *Escherichia coli,* purified and inoculated in Balb/c mice), diluted 1∶200, kindly provided by Dr. Pedro Vasconcelos from the Evandro Chagas Institute of Belém, Brazil, and by Dr. Ronaldo Borges from Federal University of Rio de Janeiro, Brazil, respectively. The specificity of anti-NS3 antibody was confirmed before with studies with DNA vaccines based on the NS3 protein and tested in mice [35].The next day, sections were incubated with a rabbit anti-mouse IgG, a secondary antibody-HRP conjugate (Spring Bioscience, CA, USA) for 30 min at room temperature. For negative control of the immunohistochemistry reaction, samples were incubated only with the secondary horseradish peroxidase-conjugated antibody. Reaction was revealed with diaminobenzidine (Dako, CA, USA) as chromogen and the sections were counterstained in Meyer’s hematoxylin (Dako).

### Electron Microscopy Assay

Tissues samples were fixed with 2% glutaraldehyde in sodium cacodylate buffer (0.2 M, ph 7.2), dehydrated in acetone, post-fixed with 1% buffered osmium tetroxide, embedded in EPON and polymerized at 60°C for 3 days. Semi-thin sections (0.5 mm thick) were obtained using a diamond knife (Diatome, Biel, Switzerland) adapted to a Reichert-Jung Ultracut E microtome (Markham, Ontario, Canada). Sections were stained with methylene blue and blue solution II [36]. Ultrathin sections (60–90 nm thick) were stained with uranyl acetate and lead citrate [37], and were observed in a Zeiss EM-900 transmission electron microscope.

### In Situ Hybridization

For *in situ* hybridization, we used one probe (5′-TGACCATCATGGACCTCCA-3′), which anneals in a conserved region inside the NS3 gene in the negative strand of viral RNA, and contained six dispersed locked nucleic acid modified bases with digoxigenin conjugated to the 5′ end. This probe was tested before in a mouse model infected with DENV, in which positive reaction was only observed in tissues from virus infected animals [31]. Paraffin-embedded sections of dengue cases and controls (5 mm) were treated for *in situ* hybridization as described elsewhere [38]. Briefly, deparaffinized sections were digested with pepsin (1.3 mg/ml) for 30 min, incubated with the probe cocktail at 60°C for 5 min for denaturation, followed by hybridization at 37°C overnight. Sections were further washed with 0.2×SSC and 2% bovine serum albumin at 4°C for 10 min. The probe-target complex was visualized due to the action of alkaline phosphatase on the chromogen nitroblue tetrazolium and bromo-chloro-indolyl-phosphate. For negative control, tissue sections of dengue cases were incubated with the solution without probe and revealed as described above.

### Double Staining for Viral RNA and Phenotypic Cell Markers

The double staining based protocol with optimization of pretreatment conditions for detection of the RNA and phenotypic markers, using *in situ* hybridization and immunohistochemistry, respectively, was previously described [39]. Briefly, the dengue probe, described above, was tagged with 5′ digoxigenin and locked nucleic acid (LNA) modified (Exiqon). The probe-target complex was visualized using an antidigoxigenin-alkaline phosphates conjugate and nitro-blue tetrazolium and 5-bromo-4-chloro-3′-indolyphosphate as the chromogen. Samples were then submitted to immunohistochemistry assay for detection of either CD68 (identification of macrophages), cytokeratin AE1/AE3 (identification of pneumocytes) and CD31 (identification of endothelial cells) and each antibody was provided in a ready-to-use form Ventana Medical Systems. Data was analyzed by the computer based Nuance system (Caliper Life Sciences, Hopkinton, MA, USA) which separates the different chromogenic signals, converts them to a fluorescent based signal and “mixes” them to determine if a given cell is co-expressing one or more target.

### Statistical Analyses

Statistical analyses were performed using Graph Pad Prism 5 software (La Jolla, CA, USA), version 4.03. Statistical differences were assessed by the Mann Whitney U test to evaluate differences in parameters between controls and DENV-patients, and values were considered significant at P<0.05.

## Results

### Liver of DENV-3 Fatal Cases: Histopathological and Ultrastructural Aspects

**Figure 1:**
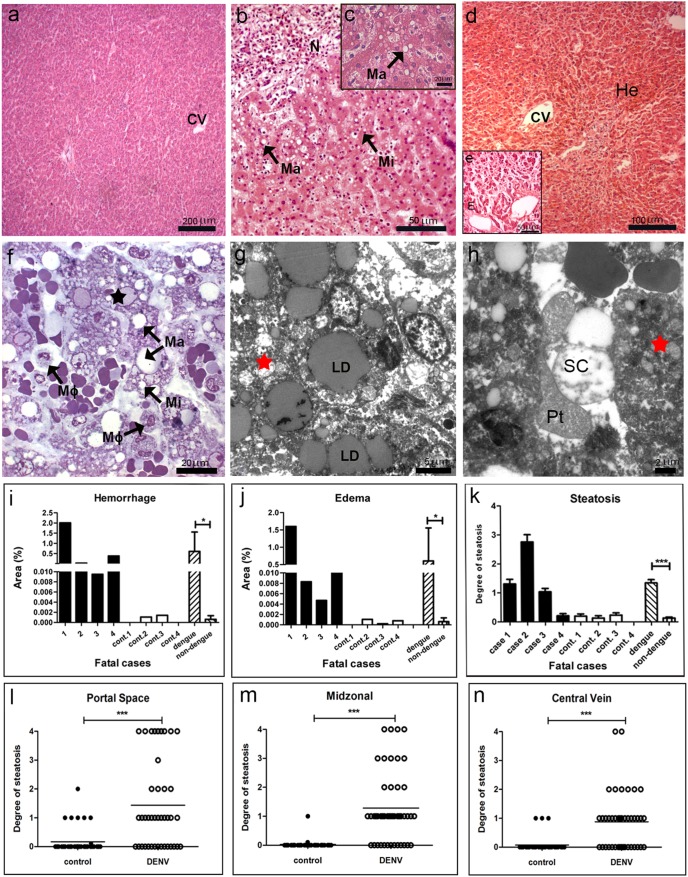
Histopathological and ultrastructural analysis of the liver. (a) Liver of a non-dengue case stained with HE and presenting normal aspect. (b–e) Liver sections of dengue cases, stained with HE, showing hepatic injuries, including micro (Mi) and macrovesicular (Ma) steatosis, necrosis (N), edema (E) and hemorrhage (He) near central vein (CV). (f) Semi-thin section of a non-dengue case presenting hepatocytes and sinusoidal capillaries with normal structures and (g) one dengue case presenting micro (Mi) and macrosteatosis (Ma), nuclear degeneration (black star) and numerous macrophage cells (Mø). (h) Ultrathin section of a non-dengue case exhibiting normal hepatocytes (H) and regular sinusoidal capillaries (SC) with the presence of monocytes (Mo) and Kupffer cells (KC) and (i and j) dengue cases showing large lipid droplets (LD) in the cytoplasm of hepatocytes, swollen mitochondria (red stars) and presence of platelet (Pt) inside sinusoidal capillaries (SC) with loss of endothelium. Semi-thin and ultrathin sections of liver were stained with methylene blue/azure II solution and uranyl acetate/lead citrate, respectively. Quantitative studies of histological damages were made individually in dengue (cases 1–4) and non-dengue patients (cont. 1–4), and statistical analysis were performed comparing the mean values of each group (dengue patients vs non-dengue patients). Damages were quantified by the percentage of affected area for (k) hemorrhage and (l) edema or (m) by steatosis degree using a scale ranging from 0 to 4. (n–o) Steatosis was also evaluated in each hepatic acini area (periportal, midzonal and central vein) by plotting different damage degrees (ten fields for each case). Asterisks indicate differences that are statistically significant between control and dengue groups, (*) (P<0.05) and (***) (P<0.0001).

Histopathological analysis of the liver showed circulatory and parenchyma damages in all studied DENV-3 fatal cases, present in all lobules. As expected, in the liver of non-dengue patients we observed a regular structure of hepatic parenchyma, with hepatocytes around the central veins and sinusoid capillaries exhibiting normal endothelial cells (Figs.1a, 1f and 1h). On the other hand, dengue cases presented severe parenchyma and circulatory alterations. The most prominent parenchyma lesion was the presence of abnormal retention of lipids inside hepatocytes with either small fat vacuoles around the nucleus (microsteatosis) or large vacuoles with displacement of the nucleus to the periphery of the cell (macrosteatosis) (Figs. 1b, 1c and 1g). Ultrastructural analysis also revealed numerous large lipid droplets inclusions inside hepatocytes, typical of macrosteatosis (Fig. 1i) and absent in the control (Fig. 1h). Quantification of steatosis revealed that three dengue cases, mainly case 2 who was obese, showed extensive areas with this damage, while case 4 presented a basal steatosis degree similar to those observed in non-dengue patients (Fig. 1m). Additionally, we observed that steatosis was present in all the three hepatic zones in the dengue cases, although it was more prominent around the portal space (Figs. 1n, 1o and 1p). Besides steatosis, the hepatic parenchyma of dengue cases also presented focal areas of necrosis with the presence of mononuclear infiltrates, mainly in case 2 (Fig. 1b). Cell death was also evidenced by detection of nuclear vacuolar degeneration in semi-thin analysis (Fig. 1g) and the presence of swollen mitochondria in ultra-thin investigations (Figs. 1i and 1j), indicating probably apoptotic processes, which were absent in the control of non-dengue patient (Figs. 1f and 1h).

Hemorrhage and edema were observed in all the four dengue cases (Figs. 1d and 1e). Quantification of these damages, detected in all the three hepatic zones with similar extensions, revealed the highest percentage in case 1, who was diabetic, followed by case 4 (Figs. 1k and 1l). We also observed the presence of numerous hyperplasic macrophages in sinusoidal capillaries (Fig. 1f) and platelets were found in the lumen of these sinusoids with concomitant loss of endothelium (Fig. 1j).

### Detection of Dengue Virus in the Liver

**Figure 2:**
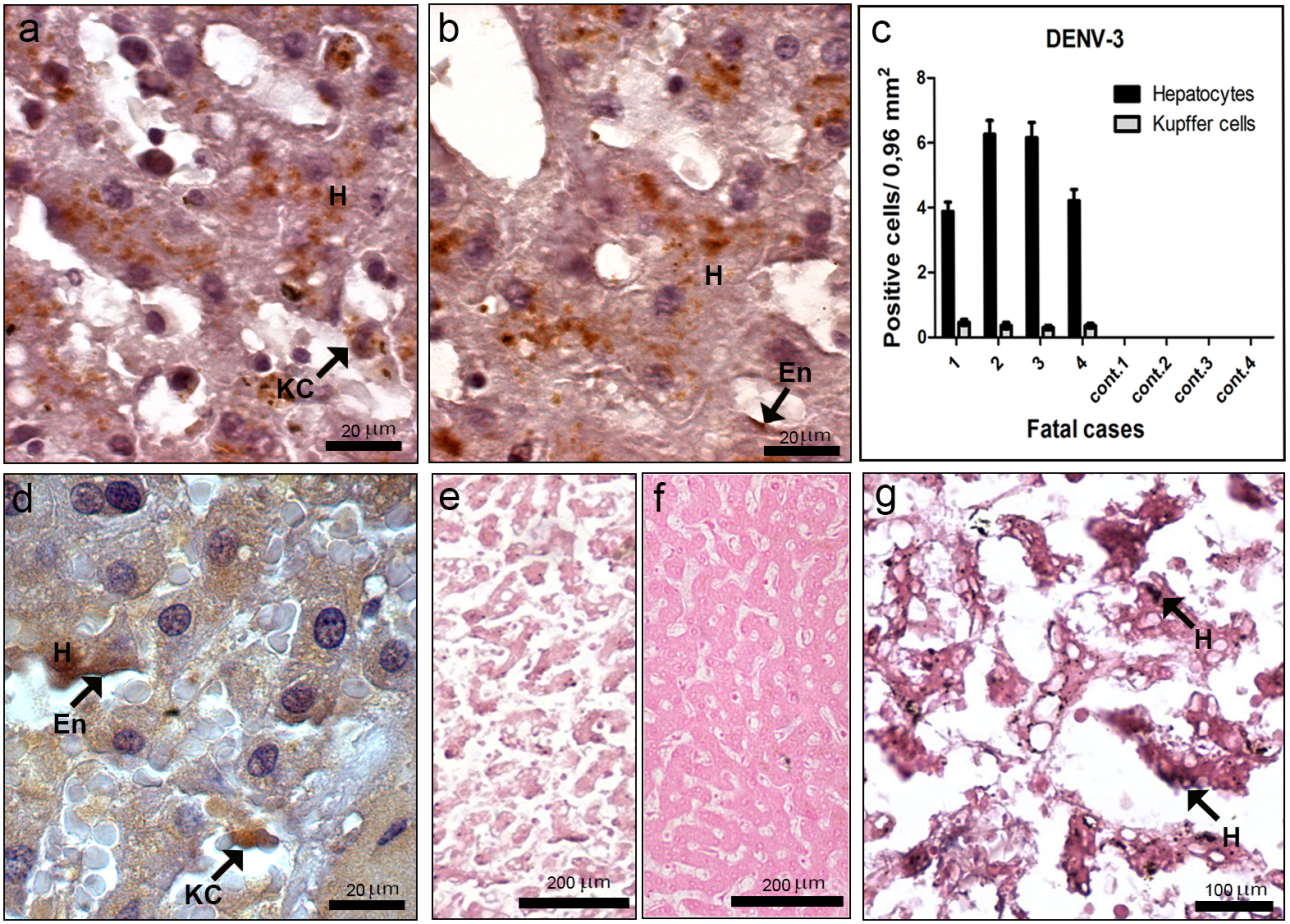
Dengue virus in the liver. (a–b) Detection of DENV-3 antigens in general, by immunochemistry, in hepatocytes (H), Kupffer cells (KC) and endothelium (En). (c) Quantification of hepatocytes and Kupffer cells presenting dengue antigens. (d) Detection of dengue NS3 protein by immunochemistry in hepatocytes (H), Kupffer cells (KC) and endothelium (En). (e–g) Detection of DENV-3 RNA negative strand by *in situ* hybridization. Dengue cases were treated without or with the probe (e and g, respectively). (f) One non-dengue case incubated with the probe. Arrows indicate positive staining in hepatocytes (H).

Initially, all cases were tested for the presence of DENV-3 antigens in general, by immunohistochemistry assay. Virus antigens were detected mainly in hepatocytes (Fig. 2a) and to a lesser extend in Kupffer cells (Fig. 2a) and in the endothelium (Fig. 2b), identified by the morphology of the cells. Virus antigens were observed only in dengue cases, in which cases 2 and 3 presented the highest number of positive cells (Fig. 2c). Virus replication in the liver was then investigated by immunochemistry assay for specific detection of the dengue NS3 protein. Results revealed that this protein was indeed present in the same cell types (hepatocytes, Kupffer and endothelial cells) containing virus antigens in general (Figs. 2a, 2b and 2d). As expected, non-dengue cases did not react with antibodies against the NS3 protein (data not shown). Additionally, the replication was also confirmed by *in situ* hybridization, using a probe that anneals only in the negative strand of the virus RNA, which revealed the presence of this RNA in hepatocytes (Fig. 2g). For negative controls, the same *in situ* hybridization assay was performed in dengue cases with omission of probe (Fig. 2e), as well as with non-dengue cases incubated with the probe (Fig. 2f), and both tests did not present positive staining.

### Lung of DENV-3 Fatal Cases: Histopathological and Ultrastructural Aspects

**Figure 3:**
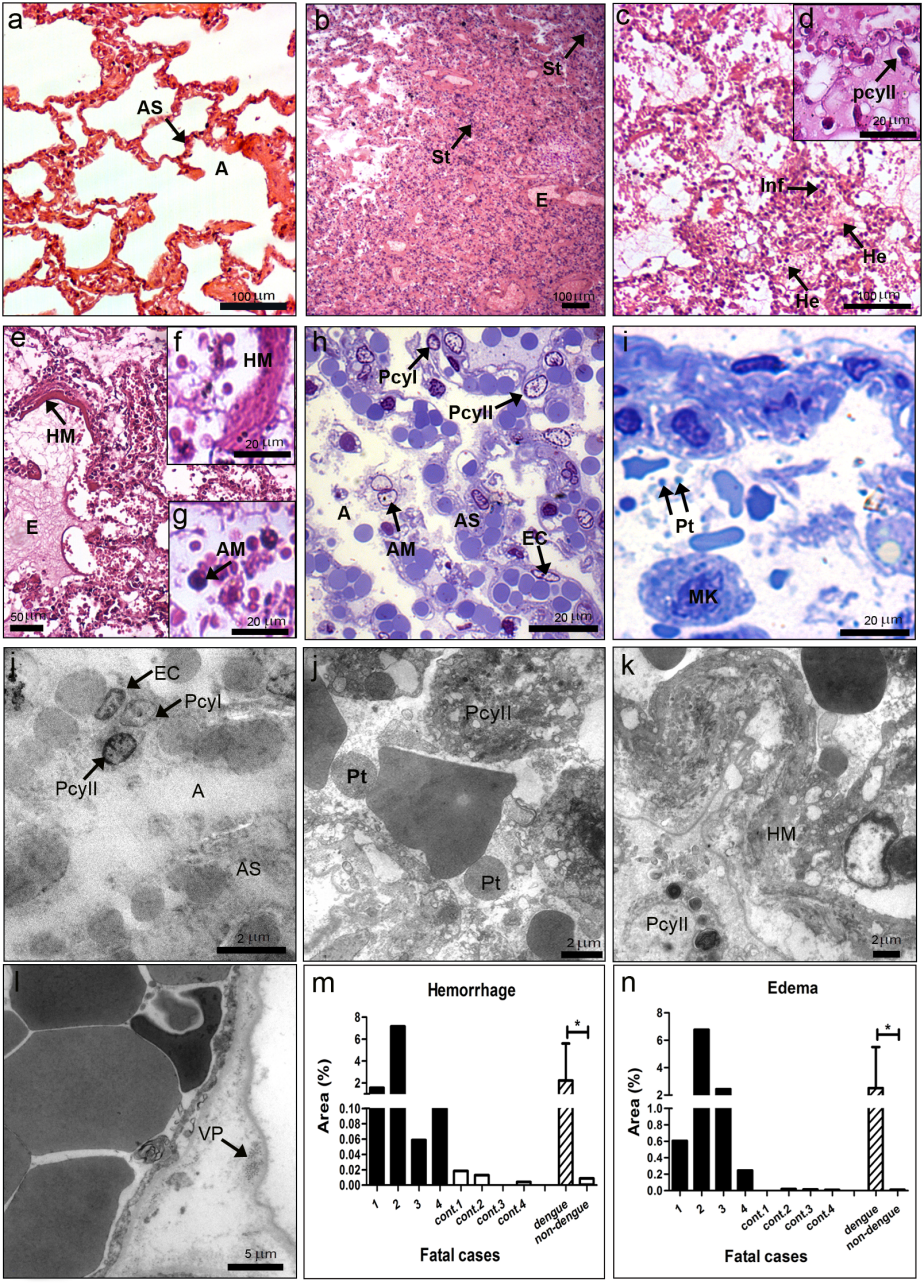
Histopathological and ultrastructural analysis of the lung. (a) Lung of a non-dengue case stained with HE and presenting normal aspect of alveoli (A) and alveolar septa (AS). (b–g) Lung sections of dengue cases, stained with HE, showing pulmonary alterations, including septal thickening (St), edema (E), hemorrhage (He), presence of mononuclear infiltrate (Inf), hyaline membrane formation (HM) and hypertrophy of alveolar macrophages (AM) and type II pneumocytes (PcyII). (h) Semi-thin section of a non-dengue case showing alveoli (A), alveolar septa (AS), endothelial cells (EC) and type I (PcyI) and II pneumocytes (PcyII) with normal aspects. (i) Semi-thin section of one dengue case presenting numerous platelets (Pt) and megakaryocytes (MK) inside alveolar septa. (j) Ultrathin section of one non-dengue case exhibiting regular alveoli, alveolar septum, type I and II pneumocytes and endothelial cell. (j-l) Ultrathin sections of dengue cases exhibited type II pneumocytes located in alveolar space in contact with numerous platelets, the appearance of hyaline membrane and the presence of virus particles (VP) in endothelium. Quantitative analysis of hemorrhage (m) and edema (n) observed in dengue (cases 1–4) and non-dengue patients (cont. 1–4), and statistical analysis performed comparing the mean values of each group (dengue patients vs non-dengue patients). Asterisks indicate differences that are statistically significant between control and dengue groups, (*) (P<0.05). Semi-thin and ultrathin sections were stained as described in figure 1, as well as damage quantifications.

Histopathological analysis of the lung showed damages in all studied DENV-3 fatal cases. As expected, we observed a regular structure of alveoli, alveolar septa and normal endothelial cells in the lung of non-dengue patients (Figs. 3a, 3h and 3j). Dengue cases presented septum thickening with an increase of cellularity (Fig. 3b), the presence of mononuclear inflammatory infiltrates (Fig. 3c) and hyperplasia of alveolar macrophages (Fig. 3g). Cases 1 and 2, who had co-morbidities (diabetes and obesity), also showed hyaline membrane formation (Fig. 3e and 3f), probably due to dengue shock syndrome, with the concomitant hypertrophy of type II pneumocytes (Fig. 3d). Alterations observed in cases 1 and 2 were evidenced with more detail in the ultrastructural analysis (Fig. 3k). Virus-like particles were also detected in the endothelium of the lung in case 1 (Fig. 3l).

All the four dengue cases presented diffuse areas with hemorrhage and edema (Figs. 3b and 3c). Quantification of these damages showed larger areas of hemorrhage in cases 1 and 2 (Fig. 3m) and edema in cases 2 and 3 (Fig. 3n). Isolated megakaryocytes and cell fragments with aspects of platelets were observed in alveolar space in semi-thin (Fig. 3i) and ultra-thin analysis (Fig. 3j).

### Detection of Dengue Virus in the Lung

**Figure 4:**
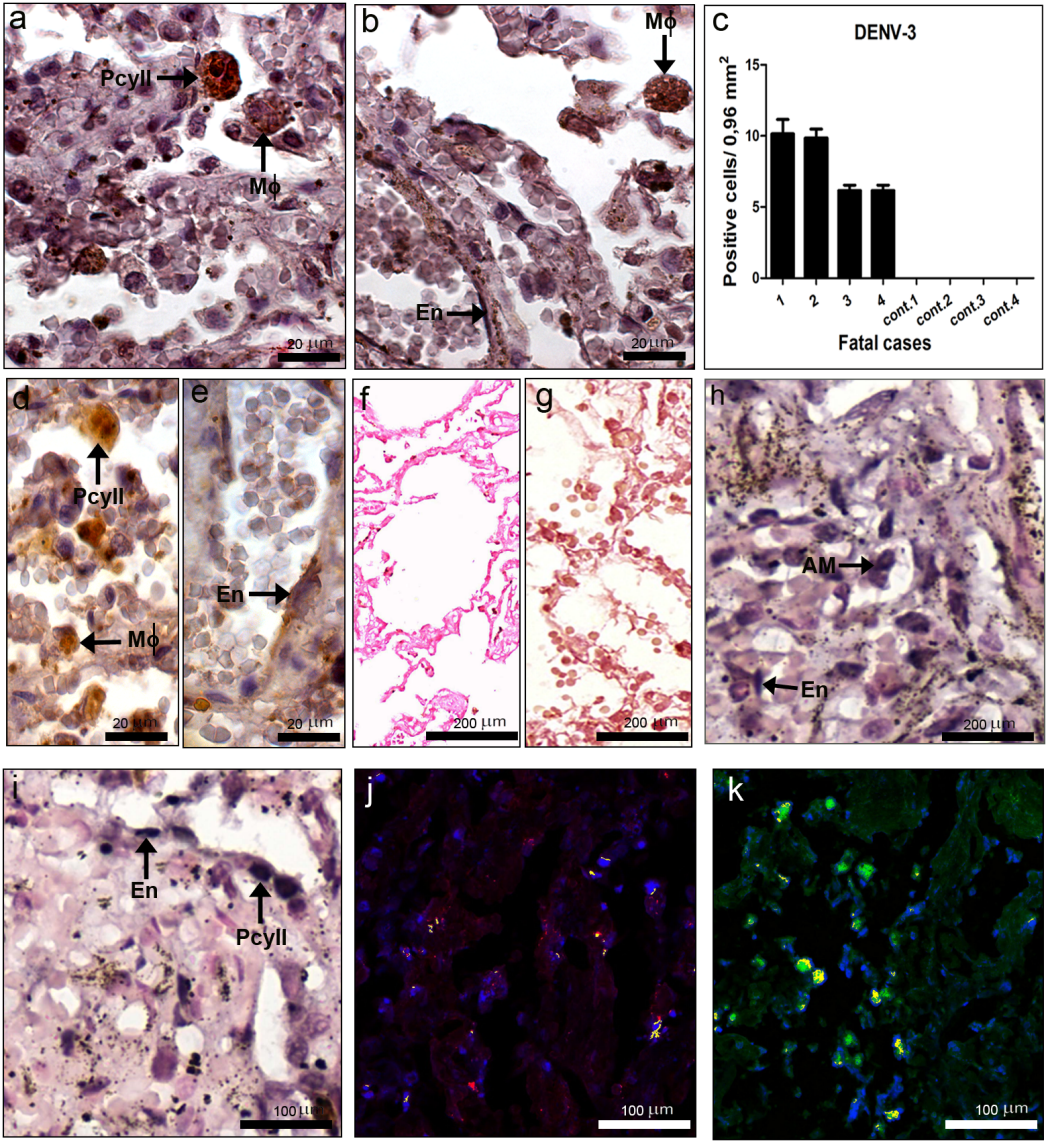
Dengue virus in the lung. (a, b, d and e) Detection of DENV-3 antigens in general (a and b) and specifically the NS3 protein (d and e), by immunochemistry, in macrophages (Mφ), type II pneumocytes (PcyII) and endothelium (En). (c) Quantification of cells presenting dengue antigens. (f–i) Detection of DENV-3 RNA negative strand by *in situ* hybridization. Dengue cases were treated without (g) or with the probe (h and i). (f) One non-dengue case incubated with the probe. (j) Co-localization of the virus RNA negative strand (fluorescent blue), by *in situ* hybridization, and CD31 (fluorescent red) for identification of endothelial cells and (k) cytokeratin AE1/AE3 (fluorescent green) for detection of pneumocytes, by immunohistochemistry. Cells presenting both staining (blue and red or blue and green) showed in yellow fluorescence.

Virus antigens were detected in the lung of all dengue cases, mainly in alveolar macrophages (Fig. 4a), but also in type II pneumocytes (Fig. 4a) and endothelium (Fig. 4b). Quantification of cells with dengue antigens revealed that cases 1 and 2 presented the highest number of positive cells (Fig. 4c). Virus replication in lung of dengue cases was observed by immunochemistry with the presence of the NS3 protein in alveolar macrophages, type II pneumocytes (Fig. 4d) and endothelium (Fig. 4e). As expected, none of lung tissues from non-dengue cases showed virus antigens (data not shown). *In situ* hybridization for detection of the dengue RNA negative strand also revealed virus replication in several alveolar macrophages, type II pneumocytes and endothelium (Figs. 4h and 4i). Dengue replication in these cells was confirmed by co-localization of the virus RNA (fluorescent blue) and CD31 (fluorescent red), for identification of endothelial cells, or cytokeratin AE1/AE3 (fluorescent green), a marker for pneumocytes (Fig. 4j and 4k). As expected, positive reaction was not observed in dengue cases treated with omission of the probe (Fig. 4g) or in non-infected tissues incubated with the probe (Fig. 4f).

### Heart of DENV-3 Fatal Cases: Histopathological and Ultrastructural Aspects

**Figure 5:**
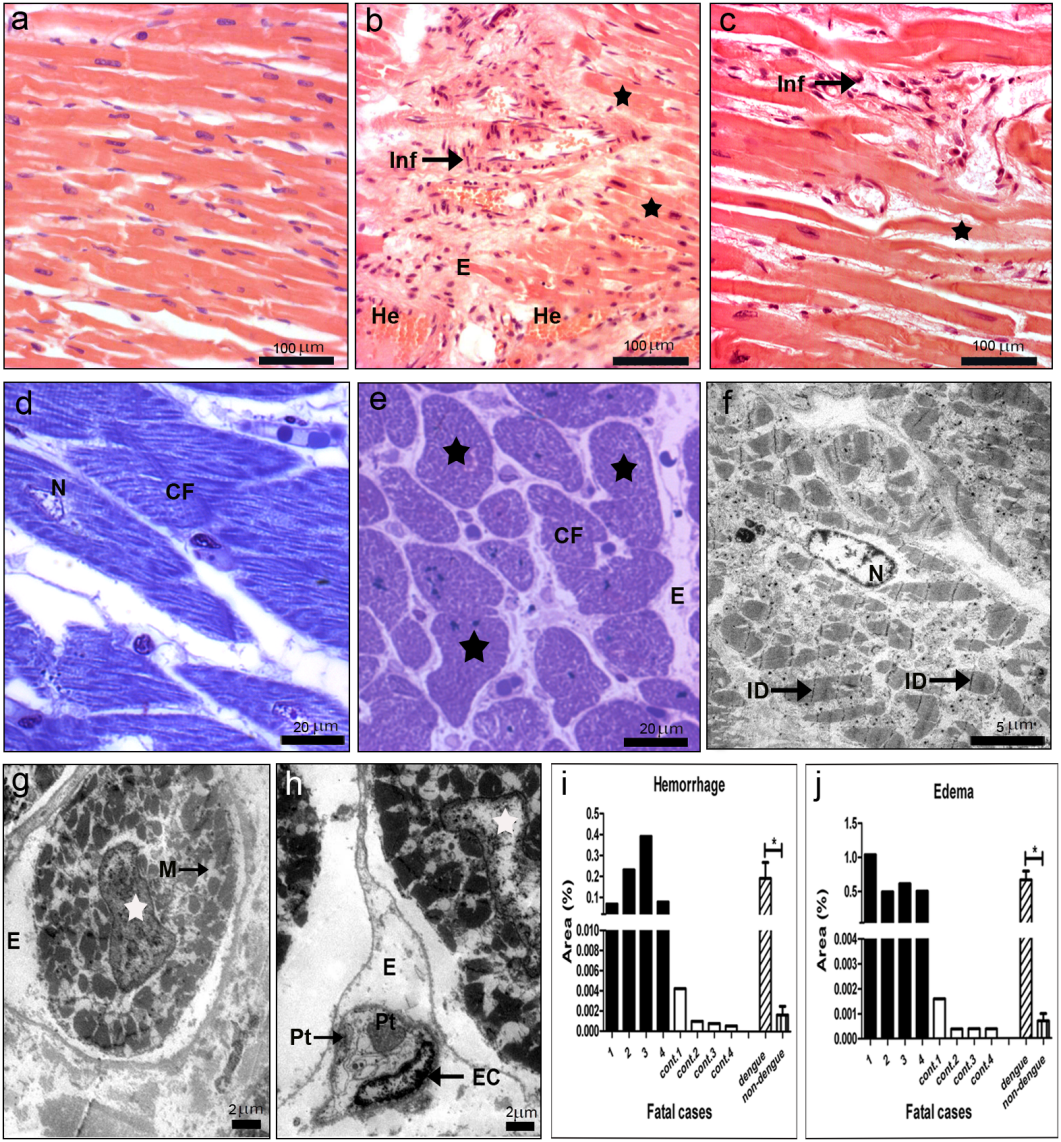
Histopathological and ultrastructural analysis of the heart. (a) Heart of a non-dengue case stained with H.E. and presenting normal aspect. (b and c) Heart sections of dengue cases, stained with HE, showing cardiac injuries, including hemorrhage (He), edema (E), presence of mononuclear infiltrate (Inf) and degeneration of muscle fibers (black star). (d and f) Semi-thin and ultrathin sections of a non-dengue case presenting cardiac fibers (CF) with normal nucleus (N), mitochondria (M), capillaries (Cap) and intercalated discs (ID). (e) Semi-thin section of one dengue case presenting degeneration of cardiac fibers (black star) characterized by absence of nucleus and a diffuse interstitial edema (E) and (g and h) ultrathin sections showing nuclear (white stars) and mitochondria alterations (M) in cardiomyocytes and interstitial edema. Quantitative analysis of hemorrhage (i) and edema (j) observed in dengue (cases 1–4) and non-dengue patients (cont. 1–4), and statistical analysis performed comparing the mean values of each group (dengue patients vs non-dengue patients). Asterisks indicate differences that are statistically significant between control and dengue groups, (*) (P<0.05). Semi-thin and ultrathin sections were stained as described in figure 1, as well as damage quantifications. (Pt) platelets; (EC) endothelial cells.

Histopathological analysis of the heart showed parenchyma and circulatory alterations in all DENV-3 cases. As expected, non-dengue patients presented a normal cardiac structure with branching fibers, central nuclei and intercalated discs (Figs. 5a, 5d and 5f). On the other hand, all dengue cases, except case 2, presented myocarditis with cardiac fibers degradation and the loss of striations and nucleus (Figs. 5b and 5c). Besides, focal areas of mononuclear infiltrated were detected in all dengue cases (Figs. 5b and 5c). Semi-thin sections revealed degeneration of cardiac fibers with the absence of nucleus and a diffuse interstitial edema, characteristic of myocarditis (Fig. 5e). Ultrastructural analysis suggested an apoptosis process in the cardiac fiber with picnotic nucleus and mitochondria alterations (Fig. 5g), which was not detected in the non-dengue patient (Fig. 5f).

Hemorrhage and edema were observed in all dengue cases (Fig. 5b). Quantification of these damages revealed that cases 2 and 3 presented extensive areas of hemorrhage (Fig. 5i), while case 1, who had diabetes and was diagnosed as having ischemic cardiomyopathy, exhibited several areas with edema (Fig. 5j). Ultrastructural evaluation also showed interstitial edema around capillary vessels in this case (Fig. 5h).

### Detection of Dengue Virus in the Heart

**Figure 6:**
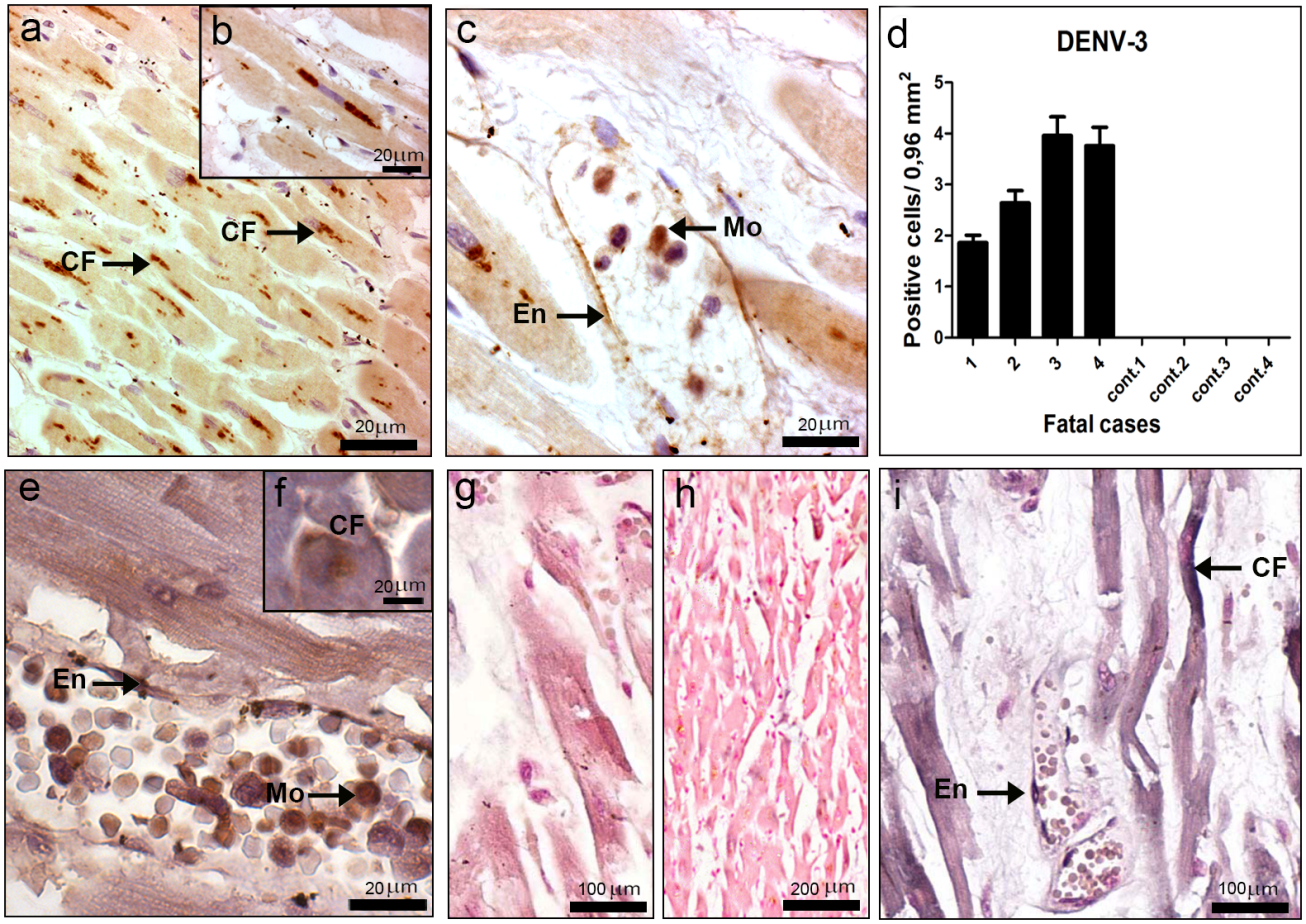
Dengue virus in the heart. (a–c) Detection of DENV-3 antigens in general (a, b and c) and specifically the NS3 protein (e and f), by immunochemistry, in cardiac fibers (CF), endothelium (En) and monocytes (Mo). (d) Quantification of cells presenting dengue antigens. (g–i) Detection of virus RNA negative strand by *in situ* hybridization. Dengue cases were treated without or with the probe (g and i, respectively). (h) One non-dengue case incubated with the probe. Arrows indicate positive staining in endothelium (En) and cardiac fibers (CF).

Virus antigens were detected mainly in the myocardial fibers in the perinuclear region (Figs. 6a and 6b), but also in monocytes/macrophages and endothelium (Fig. 6c). All dengue cases presented virus antigens, with larger detection of positive cells in cases 3 and 4, while no reaction was observed in control tissues (Fig. 6d). Virus replication in the heart of dengue cases was observed by immunochemistry with the detection of the NS3 protein in the same cell types stained for the other virus antigens (Figs. 6e and 6f). As expected, none of heart tissues from non-dengue cases showed such antigens (data not shown). The DENV replication was also confirmed by *in situ* hybridization and results revealed strong and weak positive reactions in endothelium and cardiac fibers, respectively (Fig. 6i). As expected, positive reaction was not observed in dengue cases treated with omission of the probe (Fig. 6g) or in non-infected cases incubated with the probe (Fig. 6h).

### Kidney of DENV-3 Fatal Cases: Histopathological and Ultrastructural Aspects

**Figure 7:**
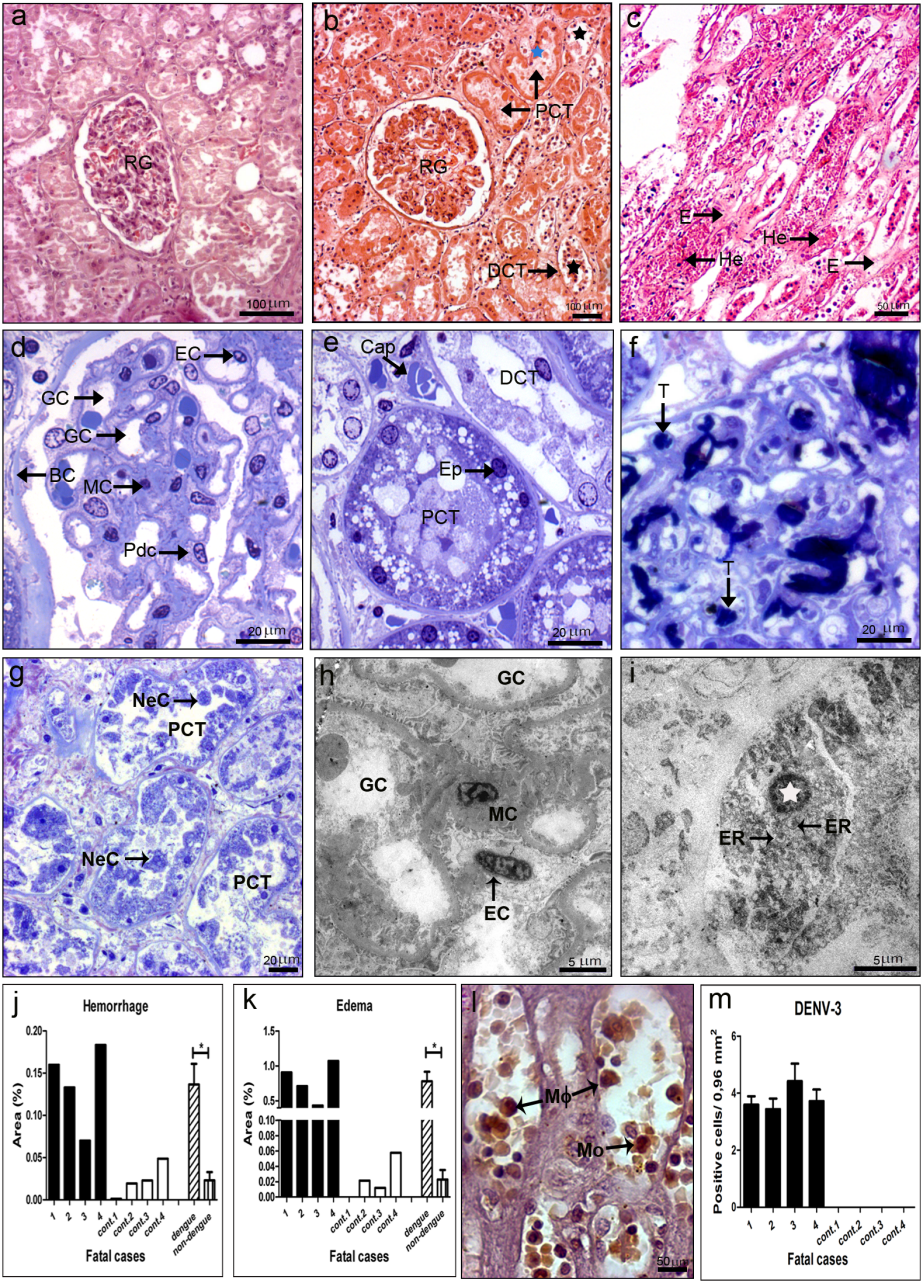
Histopathological/ultrastructural analysis and dengue detection in the kidney. (a) Kidney of a non-dengue case stained with HE and presenting normal aspect. (b and c) Kidney sections of dengue cases, stained with HE, showing injuries, including: hemorrhage (He), edema (E), sloughing of necrotic cells with loss of the basement membrane (black star), mainly in proximal convoluted tubule (PCT) but also detected in distal convoluted tubule (DCT), and areas of cellular regeneration (blue star) near renal glomerulus (RG). (d and e) Semi-thin sections of non-dengue cases showing Bowman’s capsule (BC) and podocytes (Pdc) around glomerular capillaries (GC), mensagial cells (MC) and endothelial cell (EC) with regular structures and preserved capillaries (Cap), epithelial cells (Ep) and distal and proximal convolutes tubules (DCT and PCT, respectively). (f and g) Dengue cases with the presence of thrombus (T) in capillaries of renal glomerulus and necrotic cells (NC) in the lumen of proximal convoluted tubules. (h) Ultrathin of a non-dengue case exhibiting conserved glomerular capillaries. (i) Ultrathin of one dengue case showing necrotic cell with picnotic nucleus (white star) and dilatation of rough endoplasmic reticulum (ER). Quantitative analysis of hemorrhage (j) and edema (k) observed in dengue (cases 1–4) and non-dengue patients (cont. 1–4), and statistical analysis performed comparing the mean values of each group (dengue patients vs non-dengue patients). Asterisks indicate differences that are statistically significant between control and dengue groups, (*) (P<0.05). Semi-thin and ultrathin sections were stained as described in figure 1, as well as damage quantifications. (l) Detection of DENV-3 antigens by immunochemistry in macrophages (M□) and monocytes (Mo) and (m) quantification of cells presenting these antigens.

Histopathological analysis of the kidney of the DENV-3 cases showed parenchyma and circulatory damages. As expected, in non-dengue patients we observed a normal structure with preserved distal and proximal convoluted tubules and intact renal glomerulus (Fig. 7a). Semi-thin and ultrathin sections of non-dengue cases showed podocytes around the glomerular capillaries, endothelial and mesangial cells, as well as, distal and proximal convoluted tubules presenting cuboidal cells containing brush border and capillaries vessels with regular structures (7d, 7e and 7h). In contrast, three dengue cases (1, 2 and 4) presented acute tubular necrosis, characterized by sloughing of necrotic cells and loss of the basement membrane mainly in proximal convoluted tubules, but also to a lesser extend in distal tubules, with formation of casts of cellular debris (Figs. 7b and 7g). Ultra-structural analysis revealed pyknotic nucleus and dilatation of endoplasmic reticulum in these necrotic cells (Fig. 7i). On the other hand, cellular regeneration was also found in the cortical region, indicating thus an initial recovery of the organ (Fig. 7b).

Several focal areas with hemorrhage and edema were observed in all dengue cases, located preferentially in the medullar region (Fig. 7c). Quantification of these damages showed that cases 1, 2 and 4 presented more areas with hemorrhage and edema when compared to case 3 (Figs. 7j and 7k). Circulatory disorders were also observed in semi-thin sections revealing the presence of thrombus in glomerular capillaries (Fig. 7g).

### Detection of Dengue Virus in the Kidney

Virus antigens were detected by immunohistochemical analysis, revealing that all dengue cases presented similar number of positive cells (Fig. 7m), mainly in circulating macrophages and monocytes into blood vessels (Fig. 7l). As expected, non-dengue patients did not presented virus antigens. However, no virus replication could be detected in the kidney of any of the four dengue cases, evaluated either by presence of the dengue NS3 protein or the virus RNA negative strand (data not shown).

### Spleen of DENV-3 Fatal Cases: Histopathological and Ultrastructural Aspects

**Figure 8:**
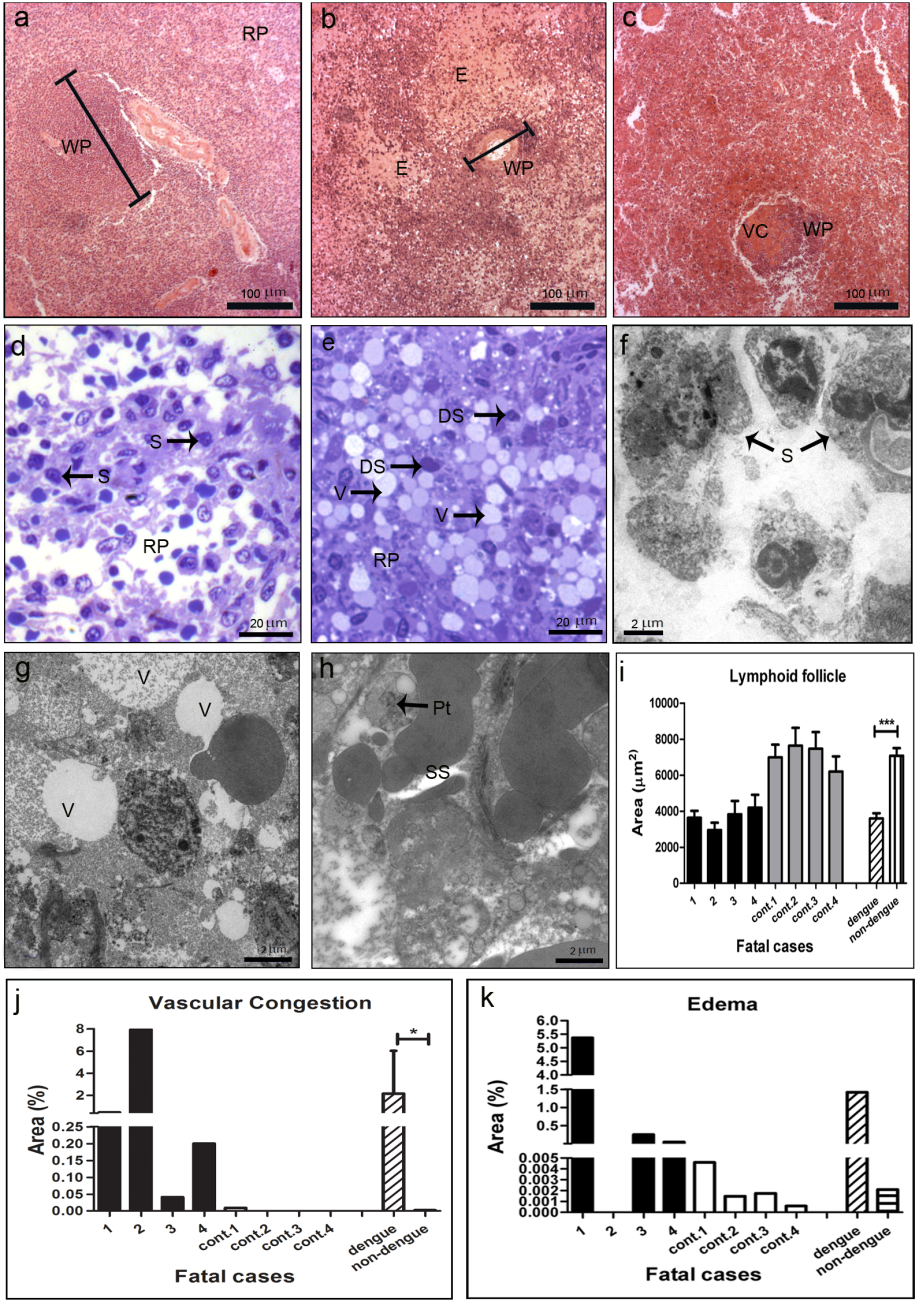
Histopathological and ultrastructural analysis of the spleen. (a) Spleen of a non-dengue case stained with HE and presenting normal aspect. (b and c) Spleen sections of dengue cases, stained with HE, showing vascular congestion (VC), edema (E) and an atrophy of lymphoid follicles. Red pulp (RP); white pulp (WP). (d) Semi-thin section of a non-dengue case revealing red pulp with regular aspect and normal splenocytes (S). (e) Semi-thin section of a dengue case showing vacuolization (V) and degenerated splenocytes (DS). (f) Ultra-thin section of a non-dengue case with regular splenocytes (S) and (g and h) dengue cases exhibiting vacuolization (V) around degenerated splenocytes and loss of the endothelium of splenic sinusoid (SS). Semi-thin and ultrathin sections were stained as described in figure 1. (i–k) Quantitative analysis of histological damages observed individually in dengue (cases 1–4) and non-dengue patients (cont. 1–4), and statistical analysis performed comparing the mean values of each group (dengue patients vs non-dengue patients). The media of lymphoid follicle areas were quantified (i), as well as the percentage areas with vascular congestion (j) and edema (k). Asterisks indicate differences that are statistically significant between control and dengue groups, (*) (P<0.05) and (***) (P<0.0001).

Histopathological analysis of the spleen of the four DENV-3 cases showed severe parenchyma and circulatory dysfunctions. Spleen of non-dengue patients presented a regular structure, with normal splenocytes and well-defined regions of white and red pulp (Figs. 8a, 8d and 8f). On the other hand, dengue cases revealed prominent parenchyma lesion with remarkable atrophy of lymphoid follicles (Fig. 8b), disruption of the structural pattern and destruction of germinal centers (Fig. 8c). Quantitative analysis of lymphoid follicle areas revealed an approximately two fold reduction of these follicles in white pulp in dengue cases when compared to non-dengue patients (Fig. 8i). Additionally, analysis of semi and ultra-thin sections revealed areas with vacuolization around degenerated splenocytes (Figs. 8e and 8g) and loss of endothelium of sinusoids (Fig. 8h), which were not observed in non-dengue case (Figs. 8d and 8f).

Several areas with vascular congestion and edema were observed in all dengue cases, located preferentially in red pulp (Figs. 8b and 8c). Quantification of these damages showed that cases 1 and 2, both with co-morbidities, had more extensive areas of congestion (Fig. 8j), while case 1 also presented more diffuse areas of edema (Fig. 8k).

### Detection of Dengue Virus in the Spleen

**Figure 9:**
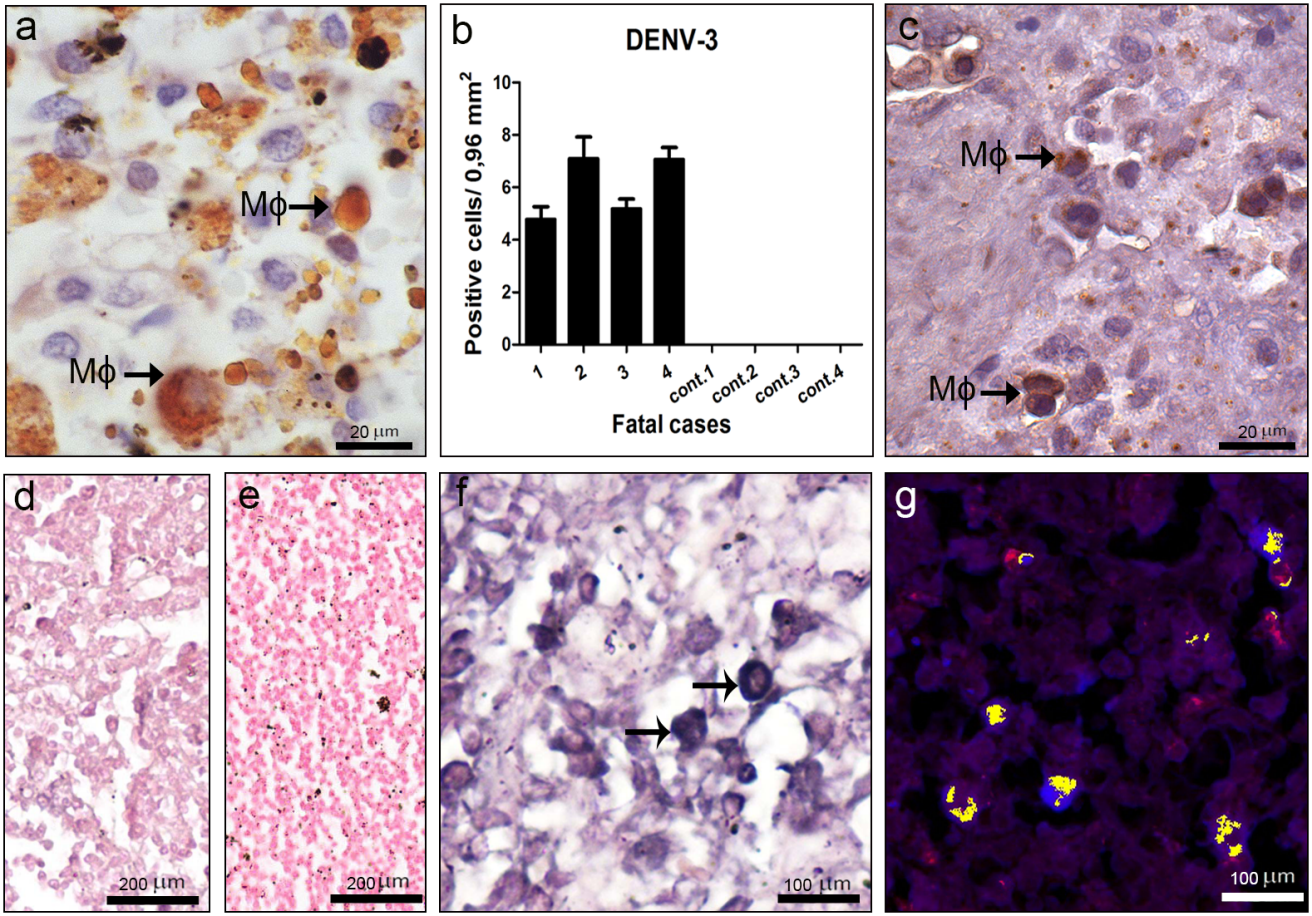
Dengue virus in the spleen. Detection of DENV-3 antigens in general (a) and specifically the NS3 protein (c), by immunochemistry in macrophages (Mφ). (b) Quantification of cells presenting dengue antigens. (d–f) Detection of virus RNA negative strand by *in situ* hybridization. Dengue cases were treated without or with the probe (d and f, respectively). (e) One non-dengue case incubated with the probe. Arrows indicate positive staining in macrophages. (g) Co-localization of the virus RNA negative strand (fluorescent blue) and CD68 (fluorescent red), for identification of macrophages. Cells presenting both staining showed yellow fluorescence.

Viral antigens were observed only in dengue cases, detected in circulating macrophages located in red pulp (Fig. 9a). Quantification of dengue positive cells showed high cell numbers in cases 2 and 4 (Fig. 9b). Dengue replication in spleen was observed in these same cells, by detection of the NS3 protein (Fig. 9c) and virus RNA (Fig. 9f). Macrophages with replicating virus were identified morphologically (Fig. 9c and 9f), as well as with the cell marker CD68 (fluorescent red), which co-localized with the dengue RNA (fluorescent blue) (Fig. 9g). As expected, reaction was not observed in tissues treated without the probe (Fig. 9d) or in non-dengue patient samples incubated with the probe (Fig. 9e).

## Discussion

Clinical observations of dengue patients and postmortem studies have provided important insights about the dengue pathophysiology, although there are still many gaps in understanding it. In the present work, we analyzed tissue samples (liver, lung, heart, kidney and spleen) from four dengue fatal cases concerning their histopathological and ultrastructural aspects, with the concomitant detection of virus in these tissues. These organs were chosen since they have been associated with dengue infection by some reports in literature [17]–[19], [23]–[27], although their involvement in the disease and death is still not clear. We observed lesions characteristic of DHF/DSS, such as hemorrhage and edema, in all organs. These observations were expected since severe dengue is usually associated to an increase of vascular permeability, which tend to lead to plasma leakage [40]–[42]. Damages, which were diffuse throughout the organs, were quantified and a particular profile of these lesions was observed in each organ.

The first organ we analyzed was the liver, which is commonly involved in dengue infection, normally leading to elevated serum levels of some hepatic enzymes [43]–[45]. We observed remarkable metabolic alterations in the hepatocytes of three DENV cases (1, 2 and 3), with the presence of single or multiple small lipid vesicles (microsteatosis) and/or large vesicles (macrosteatosis). Steatosis has been associated to dengue infection in human reports [21], [22], as well as in experimental animal models [31], [46] and *in vitro* studies [47], [48]. In fact, some studies suggested a link between lipid droplets and viral replication, with the involvement of the capsid [48] and non-structural 3 dengue proteins and increasing of the cellular fatty acid synthesis [48]. Consequently, such lipid vesicles may contribute to the spread of the virus by the hepatic tissue and subsequently to other organs. Furthermore, our analysis regarding steatosis degrees in the three hepatic zones revealed a heterogenic pattern, in which highest degrees were observed in zone I, around the portal space. This zone, described in the literature as a high oxygen content area and the first to be regenerated [49], is likewise more affected in hepatitis C cases [50], another flavivirus which seems also to use lipid vesicles for replication [51], [52]. However, in yellow fever fatal cases, Quaresma et al. [33] observed that steatosis was more intense in zone II, the midzonal area.

Quantification of liver damages comparing the four dengue cases revealed that case 2 presented the highest degree of steatosis and also focal areas of necrosis associated with mononuclear infiltrate. The existence of co-morbidity in this case, a young woman with obesity, might be one explanation for such observation. It is well known that obesity results in hepatic nonalcoholic steatosis, with mild increase of alanine and aspartate aminotransferases (ALT and AST, respectively) serum levels [53], [54]. In fact, case 2 also presented increased level of AST (149 IU/L), suggesting a liver dysfunction.

Other hepatocytes alterations, such as a vacuolar degeneration of the nucleus and the presence of swollen mitochondria, were observed in these cases by ultrastructural analysis, suggesting an apoptotic process. Liver biopsies and autopsies, obtained from either children or adults infected with DENV, also presented apoptotic cells, mainly hepatocytes and Kupffer cells [21], [22], [55]. However, evaluation of hepatic alterations by electron microscopy in dengue human cases is rare [29]. The swelling of mitochondria with the loss of its matricial chamber was also observed in dengue infected mice [30], [56] and it had been associated with alterations of the ATP balance leading to apoptosis in a hepatocyte cell line infected with DENV [46]. In fact, *in vitro* studies with several cell lines from different tissues, such as liver, lung, kidney and vascular endothelial cells, showed that dengue infection by itself can induce apoptosis [57]–[59] without the involvement of other host factors, including the various components of the immune response. However, apoptosis, as well as other damages, can be exacerbated *in vivo* by activation of inflammatory responses [15], [60].

Immunohistochemical analysis revealed the presence of dengue antigens abundantly in hepatocytes and poorly in Kupffer/macrophage and endothelial cells, although macrophages had been pointed as one of the first target cells after infection [61]. Previous studies also detected DENV antigens in hepatocytes of human dengue cases [62], [63], while other reports observed virus antigens mainly in Kupffer cells [64]. However, the existence of viral antigens, such as the envelope or membrane proteins, inside cells to assign replication may be questionable, especially in macrophages, since these antigens can be originated from phagocytized or killed virus. Therefore, we also evaluated the presence of the NS3 protein in such cells, since this is a non-structural protein which is only observed after virus replication. Results showed that this antigen was detected in the same cells (hepatocytes, Kupffer and endothelial cells) evidencing, thus, virus replication. Furthermore, we confirm these findings by *in situ* hybridization with the dengue RNA negative strand, which is only present inside cells during replication and it is a robust tool to verify virus tropism. Our results revealed strong hybridization signal in hepatocytes, thus confirming replication in these cells. Similar results were also observed in mice after dengue infection [31].

Another highly affected organ in all the four dengue cases was the lung. In fact, clinical and necropsy data, as well as our histopathological analysis, indicated that all patients died from acute pulmonary edema. Pulmonary complications during dengue infection are scarcely described, characterized mainly by hemoptysis, pulmonary hemorrhage and congestion of alveolar septa, which may lead to the rupture of alveolar walls [8], [17], [24], [65]. In the present work, we observed the highest percentage of areas with hemorrhage and edema, comparing to other organs, and several mononuclear infiltrates as well as hyperplasia of alveolar macrophages in all the four dengue cases. Furthermore, we noted that cases 1 and 2, who also had co-morbidities (diabetes and obesity, respectively), showed strong septum thickening associated with increase of cellularity. Damages in the lung tissue from these two cases indicated that they had suffered a dengue shock syndrome, which leaded to hyaline membrane formation as described elsewhere [17], [24]. The presence of hyaline membrane is also found in the lung of patients with shock in Acute Respiratory Distress Syndrome (ARDS), but its pathogenesis seems to be different from dengue, since ARDS lead mainly to neutrophil inflammation [66], [67], whereas in dengue cases we observed only mononuclear infiltrates. The abnormalities in the lung of the dengue cases were confirmed by electron microscopic. It also revealed that type II pneumocytes were highly predominant in the alveoli, mainly in case 2 and in a lesser extend in case 1, indicating that injured type I pneumocytes were removed and replaced by type II pneumocytes, which, in its turn, proliferated and became hypertrophic.

The lung was also the organ with the highest number of positive cells presenting virus antigens, thus indicating the importance of this organ in the disease, at least in severe cases. In fact, virus-like particles, detected by electron microscopy, were only observed in the lung. Dengue antigens (in general and specifically the NS3) and negative RNA strand were observed in alveolar macrophages, endothelial cells and in type II pneumocytes, indicating virus replication in these cells. Such results are in part in accordance to other findings [63], [64], which detected virus antigens in alveolar macrophages and endothelial cells. However, as far as we know, this is the first report showing virus replication also in pneumocytes. Furthermore, these results were confirmed by co-localization assays for the virus RNA and pneumocytes specific markers.

Investigation in the heart showed focal areas with mononuclear infiltrate in all the dengue cases. Moreover, in three cases (1, 3 and 4) we noted degeneration of cardiac fibers, with absence of nucleus and loss of striations, resulting from an interstitial edema, which suggests myocarditis. In fact, clinical data from case 1, who was diabetic, indicated that this patient also had cardiac problems, while necropsy data from cases 3 and 4 revealed hypertrophic cardiomyopathy. Virus antigens were detected in cardiac fibers, as well as in endothelium and monocytes/macrophages. Moreover, immunohistochemistry for detection of NS3 and *in situ* hybridization also indicated replication in cardiac fibers, which is a surprising result and confirms date from Salgado et al. [27], who detected virus antigens in these fibers. Furthermore, cardiac dysfunction had been reported in other histopathological and clinical evaluations of dengue patients [68], [69].Taken together, such findings suggest that the direct infection of the virus in cardiac fibers may be responsible, at least in part, for heart dysfunction. However, besides the direct cytotoxic effect of the virus in cardiac fibers, the exacerbation of the host immune response leading to strong cytokine expression may contribute to the observed tissue damages. Injuries seem also to involve apoptosis, which was suggested by the presence of pyknotic nuclei from cardiomyocytes and loss of mitochondrial integrity, revealed by our ultrastructural evaluations.

Regarding the kidney, the main lesion we observed was an acute tubular necrosis of proximal convoluted tubules, particularly in cases 1, 2 and 4, caused by ischemic processes, which is common in patients with severe hypovolemic shock with significant blood volume loss [70]. In this process, there is a sloughing of necrotic cells of the brush border and loss of the basement membrane in tubules with formation of casts of cellular debris. Ultrastructural analysis revealed the presence of pyknotic nuclei and dilatation of endoplasmic reticulum, suggesting the existence of booth cell death process, necrosis and apoptosis. It would be expected that case 1, who had diabetes, would present severest nephropathy with glomerular hypertrophy, indicating hyperfiltration, characteristic of this disease [71]. However, we did not observe such pathology in this case, although he exhibited elevated levels of creatinine and urea (5.0 mg/dL; and 57 mg/dL, respectively), clearly demonstrating a renal disorder. On the other hand, the dengue infection in this case potentiated vascular damages in pancreas, evidenced by the presence of arterioles with altered vascular walls and predominance of mononuclear cells (macrophages) in the midst of conjunctive tissue around the pancreatic islets (data not shown).

Interestingly, the kidney was the only organ where we did not observe DENV replication, either by detection of the NS3 protein or the virus RNA negative strand. However, we did note the presence of virus antigens in general, probably the dengue E and M proteins, in monocytes/macrophages in all studied cases. Results suggested that these antigens can be derived from immune complexes reabsorbed by such cells. Thus, these findings suggest that the kidney is not a target organ in dengue infections and that the observed injuries are not caused directly by virus replication in this organ, but due to vascular fragility probably induced by the host immune response. In fact, besides edema and hemorrhage we also observed a thrombus formation in glomerular capillaries.

On the other hand, the most remarkable effect of the dengue infection in the spleen was the atrophy of lymphoid follicles, a T and B lymphocyte rich region, observed in all the four cases, which were approximately 2 fold smaller than controls. In addition to the disruption the follicle architecture, we also noted a destruction of the germinal centers. Such results are in accordance with those from Limonta et al. [29], who reported a decrease in the amount of splenic lymphocytes in one dengue fatal case, which suggests atrophy of lymphoid follicles. Reduction of the population of TCD4+ and TCD8+ cells was also observed by flow cytometry in spleen of mice infected with DENV2 [71], [72]. As expected, virus replication was detected in circulating macrophages located in splenic red pulp of the four dengue cases, corroborating others studies [63], [64].

Overall, dengue infection involves circulatory disturbs, mainly hemorrhage and edema, described in several reports [19], [20], [40]. Besides, the histopathological analysis performed in the present work, also allowed us to identify megakaryocytes and platelets in alveolar spaces, thrombus in glomerular capillaries and loss of endothelium of splenic and hepatic sinusoids. Furthermore, we detected, by electron microscopic, platelets adhered to endothelial cells in liver, heart and spleen, which is normally not observed in health individuals. All such findings are probably due to a response to vascular damages and should play an essential role in hemostasis. Moreover, other studies showed an impaired thrombopoiesis and suppression of megakaryopoiesis in dengue patients [73], [74], [75], suggesting that infection causes extramedullar effects as a physiological compensatory mechanism that occurs when the bone marrow is unable to cover the physical demand of blood cells [75].

The vascular alterations observed in dengue cases, by its turn, may be a consequence of the imbalance of the host immune system, specially cytokine storm, cytotoxic T cell and complement activations [14], [15], [76], [77], in addition to endothelium injuries caused by the direct infection of the virus in these cells, as also reported by several *in vitro* studies [57]–[59]. Further studies will be necessary in order to investigate the contribution of the host immune response in the observed tissue damages.
